# Two-Minute Training for Improving Neonatal Bag and Mask Ventilation

**DOI:** 10.1371/journal.pone.0109049

**Published:** 2014-10-03

**Authors:** Jeroen J. van Vonderen, Ruben S. Witlox, Sascha Kraaij, Arjan B. te Pas

**Affiliations:** Department of Pediatrics, Division of Neonatology, Leiden University Medical Center, Leiden, the Netherlands; Icahn School of Medicine at Mount Sinai, Argentina

## Abstract

**Objectives:**

To test effectivity of a two-minute training consisting of a few key-points in ventilation using the self-inflating bag (SIB).

**Study Design:**

Experienced and inexperienced caregivers were asked to mask ventilate a leak free manikin using the SIB before and after the training. Mask leak and pressures were measured using respiratory function monitoring. Pressures above 35 cm H_2_O were considered excessive. Parameters were compared using a Wilcoxon non-parametric test.

**Results:**

Before and after the short training, experienced caregivers had minimal median (IQR) mask leak (14 (3-75) vs. 3 (0-53)%; p<0.01). Inexperienced users had large leak which reduced from 51 (7-91)% before to 11 (2-71)% after training (p<0.01). Pressures above 35 cm H_2_O hardly occurred in experienced caregivers (0 (0-5) vs. 0 (0-0)%; ns). In inexperienced caregivers this frequently occurred but decreased considerably after training (94 (46-100) vs. 2 (0-70)%; p<0.01).

**Conclusion:**

A two-minute training of bag and mask ventilation was effective. This training could be incorporated into any training program.

## Introduction

World-wide an estimated three to six percent of newborn infants need assisted positive pressure ventilation (PPV) at birth [1]. Adequate PPV is the cornerstone for effective neonatal resuscitation. However, achieving effective manual ventilation can be difficult [2]–[6] because most clinicians are not aware when mask leak or airway obstruction occurs and excessive peak inflation pressures (PIP) are delivered (above 35 cm H_2_O) [3], [7], [8]. The most urgent need for improving the skills of basic neonatal resuscitative actions is in low and middle-income countries where the burden of prenatal deaths and morbidity is considered to be the highest and 99% of neonatal deaths occur [1], [9].

Studies have shown that proper training significantly improves mask ventilation given by experienced and inexperienced caregivers [2], [8]. In previous studies a T-piece resuscitator (TPR) was used [2], [7], [8], which is the most used device in developed countries and is often recommended by experts [10]. However, in guidelines a self-inflating bag (SIB) and mask are also recommended for neonatal resuscitation [11]. Especially caregivers in low and middle-income countries use SIB as they have limited access to continuous gas flow [12]. The SIB is positioned horizontally and attached to the mask in a 90° angle and this could make it more difficult to create an adequate mask seal. Also, with a SIB more variable pressures are given, which can lead to either inadequate or excessive volumes delivered to the lung [13] which may cause lung injury [2]–[6], [14].

Basic neonatal skills training using basic equipment has been advocated to improve neonatal survival [15]–[17]. In line with this, repetition of ventilation skills by a simple and short mask training, emphasizing the basic essentials may contribute to improve the basic neonatal resuscitation provided. We hypothesized that when this training module is effective and not time-consuming, it could be performed regularly and incorporated in every NRP program and local training programs in all units were a SIB is used, without the necessity to make major changes.

Therefore, we tested the immediate effect of a two-minute bag and mask training by measuring mask leak and pressures given during ventilation applied by experienced and inexperienced caregivers.

## Materials and Methods

The Neonatal Intensive Care Unit (NICU) of the Leiden University Medical Centre is a tertiary level perinatal center with 400 intensive care admissions on average per year. Experienced and inexperienced caregivers (Neonatologists, fellows, registrars, midwives, neonatal and obstetric nurses) were asked to administer mask ventilation to a modified, leak-free manikin fitted with a 50 ml test lung (Modified Laerdal Resusci Baby, Laerdal, Stavanger, Norway) representing a term newborn.

PPV was applied using a Laerdal size 0/1 round mask (Laerdal, Stavanger, Norway) in combination with a Laerdal Silicone Resuscitator Pediatric Basic SIB (Laerdal, Stavanger, Norway). The Silicone Resuscitator Pediatric Basic SIB has a pressure release valve (35 cm H_2_O), but has no positive end expiratory pressure (PEEP) valve and no manometer attached. Mask leak and pressure were measured using a Respiratory Function Monitor (RFM) (Florian, Acutronic medical systems AG, Hirzl, Zürich, Switzerland). It uses a hot-wire anemometer with a dead space of 1 ml to measure gas flow in and out of the face mask. The sensor was placed between the SIB and the facemask. The flow signal was automatically integrated to provide inspired- and expired tidal volumes (V_ti_ and V_te_). Face mask leak was calculated as (((V_ti_ – V_te_)/V_ti_) * 100%). The Florian was permanently switched on during the procedure to minimize drift and the flow sensor was calibrated before each measurement. The participants were blinded to the output of the RFM.

Participants were divided in two groups, based on their experience. The experienced group consisted of neonatologists, fellows and senior registrars who received neonatal resuscitation education, including mask ventilation technique with RFM feedback, at least once during their training and applied mask ventilation in newborns on a regular basis. Experienced caregivers were included to determine if a short training was also effective for them. The inexperienced group consisted of junior registrars, neonatal and obstetric nurses who received education on neonatal resuscitation during their general training, but did not train in face mask ventilation technique with RFM feedback, and rarely used mask ventilation in a clinical setting.

Both groups performed mask ventilation on a leak free manikin for 30 s before and after training. Before training, participants were asked to administer mask ventilation at a rate of 40–60 inflations per minute using the technique they had been taught during their training [11], [18]. Participants received no further instructions but were asked to use chest excursion for evaluation of the mask ventilation given.

### The short key point training

Verbal instruction and the demonstration was standardized before commencing the study. Duration of the training has been clocked a few times during the study, which was approximately 2 minutes. All participants received a verbal instruction and demonstration by one instructor. The following key-points were explained in two minutes: 1) positioning of the head in neutral position, 2) placing of the mask on the baby's chin, rolling over mask on to the face without encroaching the eyes [19], 3) two point top hold using index finger and thumb and evenly applying pressure on the mask [19], 4) holding the ipsi or contra lateral mandible in order to squeeze the mask at the face without applying pressure on the occiput of the skull and 5) a gentle squeeze in the bag with one finger and thumb is enough to deliver appropriate tidal volumes [19] (table 1). Immediately after the instruction the second round of recordings were made, participants were not allowed to practice first and the instruction was only performed once for every single participant.

**Table 1:** Key-points discussed during face mask training using the self-inflating bag.

	Action
1	Positioning of the head in neutral position
2	Place the face mask on the baby's chin, roll it on to the face without encroaching the eyes
3	Use the two point top hold with the index finger and thumb and apply even pressure on the mask
4	Hold the ipsi or contra lateral mandible in order to squeeze the mask on the face with applying as little pressure as possible on the occiput of the skull
5	Give inflations using gentle squeeze in the bag with one finger and thumb in a rate of 40-60 inflations per minute
6	Beware that when the pop off valve of the SIB releases uncontrolled pressures can be given

Mask leak, PIP given and dispersion of pressures (expressed in SD) before and after training were analyzed and compared. The occurrence of PIP (expressed in percentage) above the limiting pressure of the pop-off valve were also calculated. As the pop-off valve was limited at 35 cm H2O we determined this as an “inadvertent pressure”, although sometimes it is possible that during neonatal resuscitation higher pressures are needed to give adequate chest rise. Comparisons between experienced and inexperienced users were not performed as this was not the aim of this study and previous studies have already reported this [8], [20], [21]. In addition, the amount of PEEP given was not measured as the SIB does not deliver PEEP.

All signals were digitized and recorded at 200 Hz using a lap-top with a data acquisition program (Spectra, Grove Medical, Hampton, England). Breaths were analyzed on a breath to breath basis.

### Ethics statement

Due to the observational character of this study the institutional review board (IRB) of our hospital (Commissie Medische Ethiek, Leids Universitair Medisch Centrum) reviewed our proposal and declared that medical ethical review of this study was not required. All staff members consented verbally to participate in this study.

### Statistical analysis

Statistical analysis was performed using SPSS (SPSS for windows, version 20.0.0, IBM, Chicago, IL, USA). Data is presented as a median (IQR), mean (SD) or percentages where appropriate. A p-value (two-sided) of <0.05 was considered to represent statistical significance. To assess the effect of training on mask leak and pressures given a Wilcoxon non-parametric test for related samples was used. Percentages for high pressures were compared using Chi square test.

## Results

During the study days a group of 52 caregivers was included of which 27 were experienced and 25 were inexperienced.

### Mask leak

**Figure 1:**
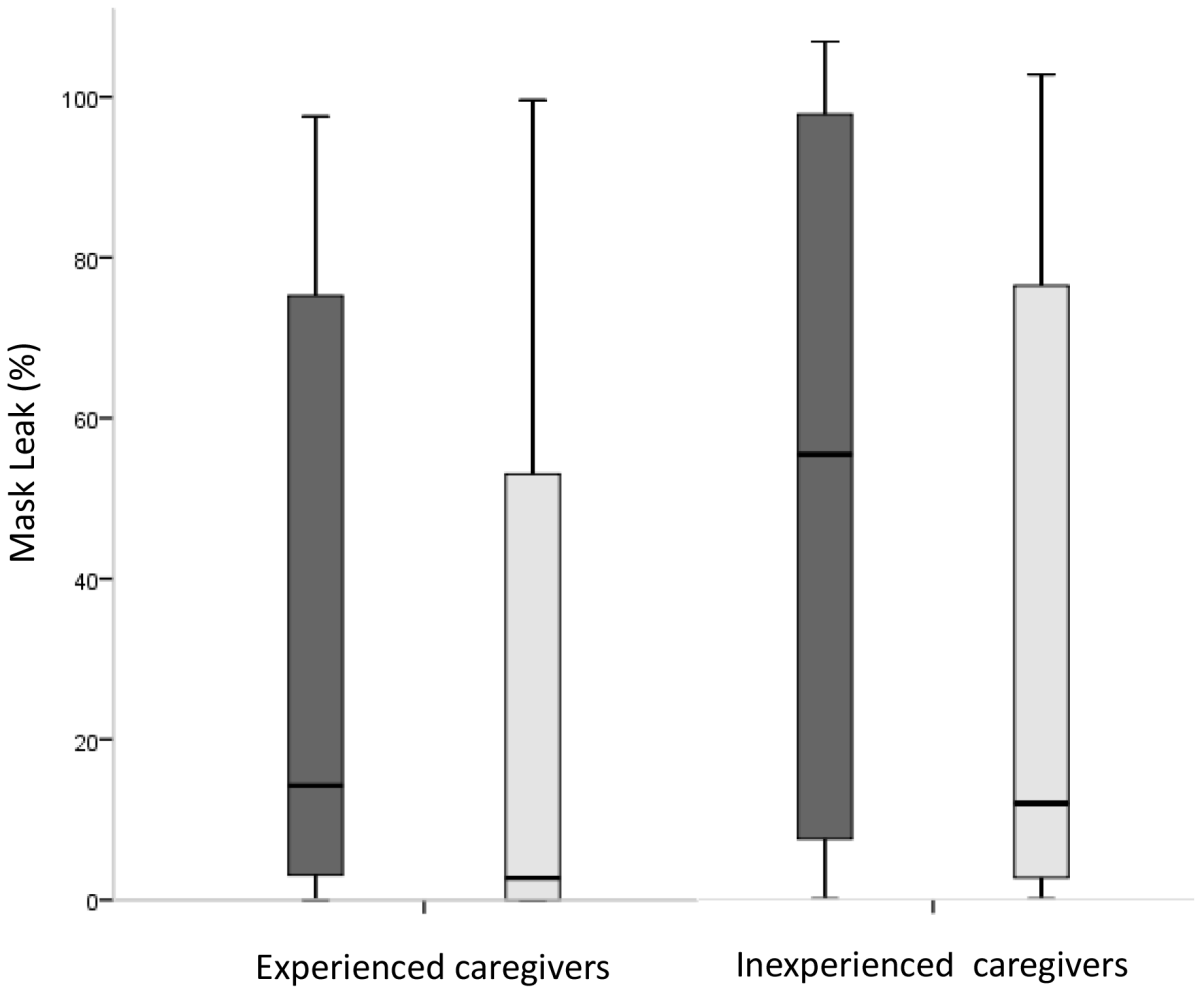
Median (IQR) mask leak (%) of experienced caregivers and inexperienced caregivers before (dark grey) and after (light grey) two-minute training using a self-inflating bag (SIB). The box plots show median values (solid black bars), IQR (margins of box), and range of data.

In the experienced group mask leak was small before training, but did decrease after training (14 (3-75)% vs. 3 (0-53)%; p<0.01) (figure 1). In the inexperienced group mask leak was large before training and significantly decreased after training (51 (7-91)% vs. 11 (2-71)%; p<0.01) (figure 1).

### Peak inflating pressure

**Figure 2:**
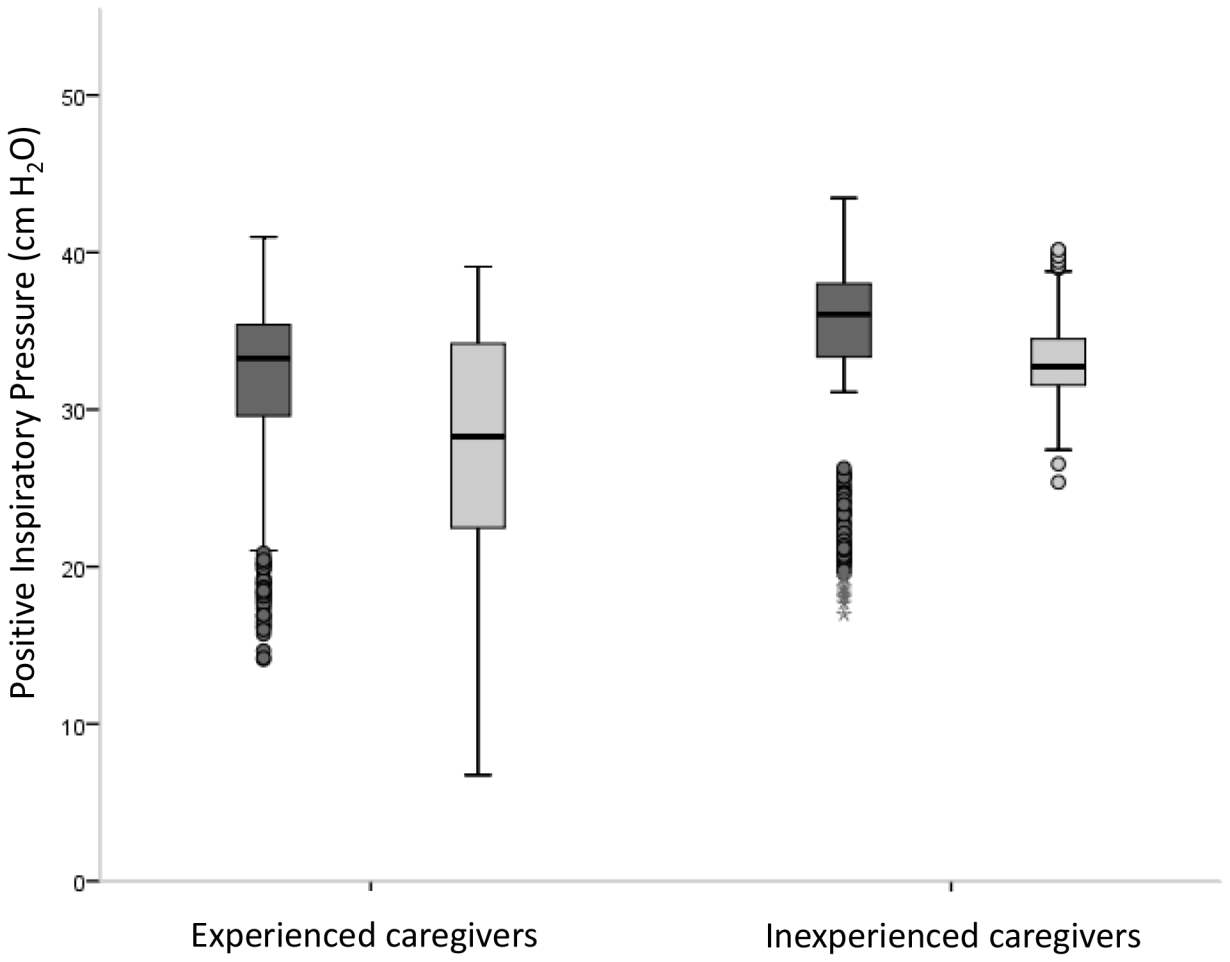
Median (IQR) pressures (cm H_2_O) given of experienced and inexperienced caregivers before (dark grey) and after (light grey) two-minute training using self-inflating bag (SIB). The box plots show median values (solid black bars), IQR (margins of box), and range of data.

Experienced caregivers delivered lower pressures after training (32 (5) vs. 28 (6) cm H_2_O; p<0.01) but pressures were not significantly different in inexperienced users (34 (6) vs. 33 (3) cm H_2_O; ns) (figure 2). Variations in pressures were unaffected by training in both experienced and inexperienced caregivers (experienced: 1.0 (0.8-1.9) vs. (1.9 (0.8-3.2) cm H_2_O; ns, inexperienced: 1.3 (1.1-1.7) vs. 1.0 (0.8-2.3) cm H_2_O; ns). Experienced caregivers very rarely delivered inflations with pressures above the pressure limit before and after training (0 (0-5)% vs. 0 (0-0)%; ns). Nearly all the inflations of the inexperienced caregivers before training were delivered with pressures above the pressure limit, but this significantly decreased after training (94 (46-100)% vs. 2 (0-70)%; p<0.01).

## Discussion

We observed that a brief instruction with key points significantly improved mask ventilation using a SIB, mask leak decreased during ventilation by both experienced and inexperienced caregivers. Although the training only had moderate effect on the median pressures given and the dispersion of pressures, inexperienced caregivers applied significantly less inflations where pressures occurred above the pressure limit after training. Experienced caregivers also significantly benefitted from training due to the significant decrease in leak. Since the instruction was short and simple, the two minutes bag and mask training could easily be incorporated in existing training modules.

Previous studies [2], [8], [22] with more elaborate training have shown a decrease in mask leak after training of mask technique. In the study of Wilson et al. training was more effective in reducing the amount of leak compared to our study [22]. However, we demonstrated that a short training of a few key points also had a significant effect on mask technique. Most mask leak training studies were performed using a TPR [2], [8]. However, we were interested in improving mask leak and inadvertent pressures when using the SIB. Although it has been shown that the TPR is the best available device to deliver accurate and consistent pressures [20], [23], many hospitals in developed and developing countries use the SIB for neonatal resuscitation. Although dispersion of pressures remained similar, the short training reduced the risk for excessive pressures.

Giving mask training that is short and simple, with the emphasis on key points, is in line with the current concept of using simplified versions of neonatal resuscitation in developing countries [15]. Although our previous study showed that effect of mask training was long lasting [8], it is possible that this does not account for a 2 minute training. Although experienced caregivers, who previously have been shown to benefit less from training [8], leak was significantly decreased after this short training. Mduma et al. have shown in their low dose high frequency training program “helping babies breathe” that frequent re-training improved skills and clinical practice [24]. It is likely that frequent repetition of the two minute mask training should be taken into account when incorporating this into the simulation-based training programs.

We observed that despite the presence of a pop-off valve on a self-inflating bag it was possible to give pressures above the supposed 35 cm H_2_O limit. This is not a new finding and has been reported in a previous study [20]. The higher pressures are explained as a result of a combination of ventilation rate and inertia of the pop-off valve, the inspiration time is shorter than the time needed for the valve to open [20]. However, the higher pressures did hardly occur in experienced caregivers. It is possible that there is more awareness among experienced caregivers of the danger in delivering high pressures and even before training effort has been put in avoiding this. In contrast, the occurrence of high pressures was very high in inexperienced users. The short instruction made the occurrence of high pressures almost disappear.

This study was performed in a center where mask training using a leak-free manikin and RFM is incorporated in the neonatal resuscitation training. It is possible that this could have led to a bias and possibly different results would be reached in other centers. However, this could only be the case for the experienced caregivers and this could have led to better results. The use of a leak-free manikin and RFM as direct feedback during these kind of studies and trainings is important as it is difficult to monitor leak and pressures (if the pop off valve is not clearly triggered) when using the SIB. The RFM has recently been recommended for mask ventilation training [7], [8], but unfortunately the device is not widely available. Although in this study a RFM has been used, we blinded the caregivers for the RFM and comparable results could be expected when training is given without a leak free manikin and direct feedback. Therefore the training (without a RFM) could be performed in low resource settings as well as in other units were a SIB is used. The aim of our training was to reduce mask leak and the frequency of inadvertent pressured. Ventilation rate, which is also an important part of the neonatal resuscitation, was not recorded in this study. However, as leak and occurrence of inadvertent pressures decreased significantly the same effect can be expected of achieving the correct ventilation rate.

In conclusion, during neonatal bag and mask ventilation mask leak is small when applied by experienced caregivers, but is large when it is given by inexperienced caregivers. A two-minute training with a few key points significantly decreased mask leak to acceptable levels in both experienced and inexperienced caregivers. The training had no influence in the inconsistency of the given pressures. However, the amount of high pressures given by inexperienced user, for which the pop-off valve apparently does not protect, considerable decreased after training. To improve immediate ventilation skills, this short and simple bag and mask training could easily be incorporated in training programs.
